# Frequency of Antimicrobial-Resistant Fecal *Escherichia coli* Among Small, Medium, and Large Beef Cow–Calf Operations in Florida

**DOI:** 10.3390/microorganisms14010013

**Published:** 2025-12-20

**Authors:** Ahmad Ali, João H. J. Bittar, Lekshmi K. Edison, James Colee, Thomas Denagamage, Jorge A. Hernandez, Subhashinie Kariyawasam

**Affiliations:** 1 Department of Comparative, Diagnostic, and Population Medicine, College of Veterinary Medicine, University of Florida, Gainesville, FL 32608, USA; aliahmad@ufl.edu (A.A.); edison.le@ufl.edu (L.K.E.); 2 Department of Medicine, Cholistan University of Veterinary and Animal Sciences, Bahawalpur 63100, Pakistan; 3 Department of Large Animal Clinical Sciences, College of Veterinary Medicine, University of Florida, Gainesville, FL 32608, USA; jbittar@ufl.edu (J.H.J.B.); tdenagamage@ufl.edu (T.D.); hernandezja@ufl.edu (J.A.H.); 4 Statistics Consulting Unit, Institute of Food and Agricultural Sciences (IFAS), University of Florida, Gainesville, FL 32611, USA; colee@ufl.edu

**Keywords:** antimicrobial resistance, *Escherichia coli*, beef cow-calf operations, operation size

## Abstract

Antimicrobial resistance (AMR) is a growing threat to both animal and human health. This study investigated the occurrence of AMR in *Escherichia coli* isolated from fecal samples collected from beef cow–calf operations and compared the frequency of antimicrobial-resistant fecal *E. coli* among small, medium, and large beef cow–calf operations in Florida, United States. The study included nine beef cow–calf operations. Between December 2023 and April 2024, a total of 743 fecal samples were collected from cows (*n* = 429) and calves (*n* = 314), either directly from the rectum or from fresh voided feces. From these samples, 3475 *E. coli* isolates (five isolates/animal) were subjected to antibiotic susceptibility testing using the Kirby–Bauer disk diffusion method. A panel of eight antibiotics was used to assess AMR profiles. Irrespective of farm size, cows and calves showed higher resistance to streptomycin (47% or 330/695), oxytetracycline (46% or 319/695), sulfadimethoxine (42% or 291/695), ampicillin (41% or 283/695), and florfenicol (18% or 126/695). In contrast, lower resistance frequencies were observed for gentamicin (4% or 27/695), ceftiofur (3% or 19/695), and trimethoprim–sulfamethoxazole (3% or 18/695). Animals from medium (OR = 1.67) and large operations (OR = 2.70) had greater odds of harboring *E. coli* resistant to ≥1 antibiotic than those from small operations. Streptomycin (medium OR = 1.92; large OR = 4.17) and sulfadimethoxine resistance (medium OR = 1.64; large OR = 3.45) were also more frequent in medium and large operations, respectively. Additionally, *E. coli* resistance to florfenicol was higher in calves (OR = 3.57) than in cows, after controlling for farm size. This study provides new insights into AMR patterns in fecal bacteria from beef cow–calf operations in Florida and can help producers and veterinarians develop informed strategies for monitoring and mitigating AMR.

## 1. Introduction

Antimicrobial resistance (AMR) is a major concern in cattle production systems because AMR pathogens can lead to increased morbidity and mortality in livestock, resulting in greater treatment and production costs for producers [1]. From a public health perspective, AMR pathogens can also spread to humans, either directly through contact with animals [2] or indirectly via the food chain [3,4]. The World Health Organization (WHO) has recognized AMR as one of the greatest threats to global health in the 21st century [5]. It is estimated that, without effective interventions, AMR could cause up to ten million mortalities annually by 2050, with substantial economic losses projected to rise dramatically by mid-century [5,6].

The use of antibiotics is well recognized as a major driver of AMR, but resistance can also develop in the absence of direct antibiotic exposure [7]. Generic *Escherichia coli* is widely used as an indicator organism for monitoring AMR trends because it is a commensal inhabitant of the bovine gastrointestinal tract and a potential reservoir of resistance genes that can be transferred to other bacteria [8,9]. On a global scale, relatively few studies have focused on AMR in beef cow–calf operations [10]. Most studies have focused on feedlots, stockers, and calf ranch operations, where bacterial populations may experience elevated selection pressures due to the use of antimicrobials for therapeutic purposes. These pressures are compounded by management-related factors such as stress, transportation, and animal mixing [8]. In beef cow–calf operations, calves are born and raised alongside their dams at the same location until approximately 6–8 months of age. At this time, they are then weaned, separated from their dam, and grouped with other animals of similar age and/or weight. After weaning, most calves are transferred to feedlots, where they remain until they reach slaughter weight. However, adult cull cows from cow–calf operations are typically sent straight to slaughter at the end of their productive lives [8]. Overall, very few studies have evaluated AMR in beef cow–calf operations globally, and such studies are particularly rare in the United States, as noted in recently published literature [10]. Despite the importance of cow–calf operations as the primary source of calves entering the beef industry, limited research has assessed AMR at this stage, especially operations of different sizes. Consequently, the relationship between farm size, animal class (cow versus calf), and the prevalence of antimicrobial-resistant *E. coli* remains largely unknown.

In United States beef cow–calf systems, antimicrobial use is relatively limited, compared with feedlot production. This is particularly evident following FDA policy changes, implemented in 2017, that eliminated the use of medically important antibiotics for growth promotion and required veterinary oversight for their use in feed or water. According to a USDA report, the primary antimicrobial classes used in cow–calf herds include tetracyclines, macrolides, aminoglycosides, β-lactams, and sulfonamides [11]. These drugs are mostly administered to treat or control conditions such as respiratory disease, pinkeye, foot rot, lameness, and neonatal diarrhea. Injectable and oral treatments are more common than feed additive products, and their use generally increases with herd size. The most frequently reported antibiotics used in cow–calf operations are oxytetracycline, tulathromycin, penicillin, florfenicol, and ceftiofur, as mentioned in a previous study [12] and national report [11]. Several of these antimicrobial classes, including macrolides and third-generation cephalosporins, are considered critically important for human medicine and, therefore, fall under strengthened national regulatory oversight. This context is essential for interpreting antimicrobial resistance patterns in cow–calf operations and highlights the need for additional research in this underrepresented production sector.

Florida is predominantly a cow–calf state, ranking tenth nationally in beef cattle production, with approximately 865,000 heads of beef cows [13]. Understanding and characterizing AMR in these operations is crucial for evaluating the contribution of the cow–calf sector to AMR concerns in the broader beef industry. Previous studies conducted in the Western United States (California, Oregon, and Washington) and Florida have indicated that management- and operation-dependent factors, including operation size and animal type, can influence the prevalence of AMR [12,14]. Based on this, we hypothesized that antimicrobial-resistant *E. coli* would be more prevalent in medium and large beef cow–calf operations, compared with small operations, and that calves would exhibit higher levels of resistance than cows. Therefore, the primary objective of this study was to compare the frequency of fecal *E. coli* resistant to selected antibiotics in beef cattle housed in small, medium, and large beef cow–calf operations. A secondary objective was to compare the frequency of cows and calves harboring fecal *E. coli* resistant to selected antibiotics.

## 2. Materials and Methods

### 2.1. Study Cow–Calf Operations

A convenience sample of nine beef cow–calf operations in Florida, United States, were enrolled in this study. Operations were categorized by size: small (20–49 cows; *n* = 3), medium (50–199 cows; *n* = 3), and large (≥200 cows; *n* = 3) [11]. The nine enrolled beef cow–calf operations were distributed across five Florida counties: Okeechobee, Hardee, Marion, Pasco, and Sumter. In Florida, beef cow–calf operations typically raise cattle under extensive, free-range conditions. Warm-season grasses serve as the primary forage base, often supplemented with minerals. During the winter, additional nutrition is provided through hay, concentrate supplements, or grazing on winter annuals. These region-specific feeding and management practices, combined with the subtropical climate, may influence cattle health, antimicrobial use, and, consequently, patterns of antimicrobial resistance [12]. All farms adhered to herd-specific vaccination and deworming programs under the guidance of their veterinarians. The study protocol was approved by the IACUC (protocol #202300000165) at the University of Florida.

### 2.2. Study Animals

A total of 97, 213, and 433 beef cattle (cows and calves) were selected from three small, three medium, and three large beef cow–calf operations, respectively, during the period between 14 December 2023 and 4 April 2024. The cows ranged in age from 14 months to 10 years, and calves from 1 month to 8 months. The predominant breeds were Brangus, Hereford, Red Angus, and various crossbreeds commonly found in Florida beef herds. All animals appeared to be healthy at the time of sampling. The number of cattle selected from each study farm was justified using the following assumptions: the expected prevalence of AMR fecal *E. coli* was set at 50 ± 10% for large beef cow–calf operations, 25 ± 10% for medium-sized operations, and 12.5 ± 10% for small beef cow–calf operations, with a confidence level of 95%. The prevalence of 50%, 25%, and 12.5% was based on a previous study [15]. Table 1 shows the number of animals sampled in each beef cow–calf operation.

### 2.3. Collection of Fecal Samples

Fecal samples were collected either directly from the rectum of each animal or from freshly defecated feces, using individual rectal palpation sleeves or gloves to avoid cross-contamination. Samples were collected in sterile bags, labeled, and transported on ice to the laboratory at the College of Veterinary Medicine, University of Florida. Samples were processed and cultured within 24 h of collection for *E. coli* isolation. All *E. coli* isolates were stored at −80 °C in 25% glycerol for subsequent analysis.

### 2.4. Isolation of Fecal E. coli

Fresh fecal samples were homogenized in approximately 2 mL of sterile phosphate-buffered saline (PBS). A sterile cotton swab was inserted into the fecal homogeneate and streaked onto *E. coli* ECD ChromoSelect agar with MUG (MilliporeSigma, Merck KGaA, Darmstadt, Germany), following the manufacturer’s guidelines. The plates were incubated for 18–24 h at 44 °C and presumptive *E. coli* colonies were identified based on their characteristic blue-green color. Colonies were confirmed as *E. coli* by a positive indole spot test (Remel, Lenexa, KS, USA). Five discrete *E. coli* colonies from each sample plate were subcultured on MacConkey agar (Becton Dickinson and Company, Franklin Lakes, NJ, USA) and incubated at 37 °C for 24 h. Pink colonies from each plate were grown individually in Luria–Bertani (LB) broth at 37 °C and cultures were stored at −80 °C in LB broth containing 25% glycerol in 2 ml cryovials until use.

### 2.5. E. coli Isolate Selection

Following the initial culture of 743 fecal samples on ChromoSelect agar, 48 samples were excluded because they yielded no *E. coli* colonies or fewer than five discrete colonies. For antimicrobial susceptibility testing (AST), a total of 3475 isolates (five isolates per animal) obtained from 695 cow and calf fecal samples were selected. Table 1 presents the numbers of cows and calves included in the AST.

### 2.6. Antimicrobial Susceptibility Testing (AST)

The antimicrobial susceptibility profiles of *E. coli* were determined using the Kirby–Bauer disk diffusion method [16]. Each isolate was tested against a panel of eight antibiotics representing major antimicrobial classes commonly used in beef cow–calf operations. Selection of these antibiotics was guided by previous literature [11,12,17] and informal information gathered from ranchers regarding commonly used therapeutic practices. These antibiotics included penicillins (ampicillin, 10 µg), third-generation cephalosporins (ceftiofur, 30 µg), amphenicols (florfenicol, 30 µg), aminoglycosides (gentamicin, 10 µg; streptomycin, 10 µg), tetracyclines (oxytetracycline, 30 µg), trimethoprim–sulfonamide combination (trimethoprim/sulfamethoxazole, 1.25/23.75 µg), and sulfonamides (sulfadimethoxine, 300 µg). Antibiotic discs of standard concentrations were prepared in our laboratory as previously described [18], except for ceftiofur, for which we used commercially available discs (Becton Dickinson and Company, Franklin Lakes, NJ, USA). The diameter of the zone of inhibition was measured using a Vernier caliper and interpreted according to CLSI guidelines [19] or previous publications, when CLSI breakpoints were unavailable [20]. *E. coli* ATCC^®^ 25922 and *E. coli* ATCC^®^ 35218 were included as the reference strains and tested concurrently with study isolates to ensure accuracy and reproducibility of results. An individual animal (cow or calf) was classified as carrying resistant *E. coli* if at least one of the five isolates obtained from that animal was resistant to one or more antibiotics. Animals were classified as carrying multi-drug-resistant (MDR) *E. coli* if at least one isolate was resistant to three or more antimicrobial classes [21].

### 2.7. Data Analysis

A descriptive analysis was performed in Microsoft Excel to determine the frequency (%) of antimicrobial-resistant *E. coli*. The following null hypotheses were tested.

**H01.**  *There is no difference in the frequency of beef cattle with E. coli resistance to selected antibiotics housed in small, medium, and large beef cow–calf operations.*

**H02.**  *There is no difference in the frequency of cows and calves diagnosed with E. coli resistance to selected antibiotics.*

A generalized linear mixed model (GLMM) with a binary distribution and logit link function was used to estimate the odds of antimicrobial resistance. Fixed effects included farm size and animal type, while herd was included as a random effect to account for clustering within herds. Data were summarized as counts of resistant and non-resistant, per farm size and animal type. Empirical (robust) standard errors were applied to address overdispersion in the data. Pairwise comparisons among significant fixed effects were adjusted using Tukey’s multiple comparison test, unless otherwise noted. Odds ratios (OR) and 95% confidence intervals (CI) were derived from the model estimates. For ceftiofur, the model did not converge due to the low number of resistant isolates. All analyses were performed in SAS version 9.4 (SAS Institute Inc., Cary, NC, USA).

## 3. Results

### 3.1. Isolation of E. coli from Fecal Samples

*E. coli* was recovered from 94% (695/743) of fecal samples collected from cows and calves across nine beef cow–calf operations. A total of 3475 *E. coli* isolates (five isolates/animal) from cows and calves were subjected to AST.

### 3.2. Frequency of Beef Cattle with E. coli Resistance to Selected Antibiotics Housed in Small, Medium, and Large Beef Cow–Calf Operations

**Figure 1:**
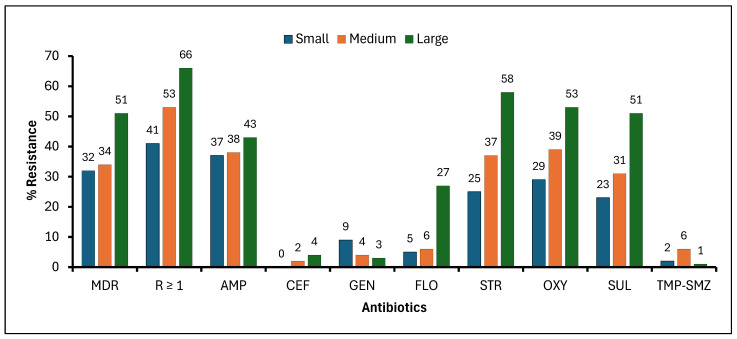
Frequency of fecal *Escherichia coli* resistance to eight antibiotics among beef cattle from small, medium, and large beef cow–calf operations. (MDR = Multi-drug resistance; R ≥ 1 = Resistance to one or more antibiotics; AMP = Ampicillin; CEF = Ceftiofur; GEN = Gentamicin; FLO = Florfenicol; STR = Streptomycin; OXY = Oxytetracycline; SUL = Sulfadimethoxine; TMP-SMZ = Trimethoprim–sulfamethoxazole).

Overall, resistance to one or more antibiotics was observed in 59% (412/695), while multi-drug resistance was detected in 44% (304/695) of animals. The highest frequency of resistance was observed for streptomycin (47% or 330/695), followed by oxytetracycline (46% or 319/695), sulfadimethoxine (42% or 291/695), ampicillin (41% or 283/695), or florfenicol (18% or 126/695). The lowest resistance frequencies were observed for gentamicin (4%, or 27/695), ceftiofur (3%, or 19/695), or trimethoprim/sulfamethoxazole (3%, or 18/695) (Figure 1 and Table S1).

### 3.3. Frequency of Cows and Calves Diagnosed with E. coli Resistance to Selected Antibiotics

**Figure 2:**
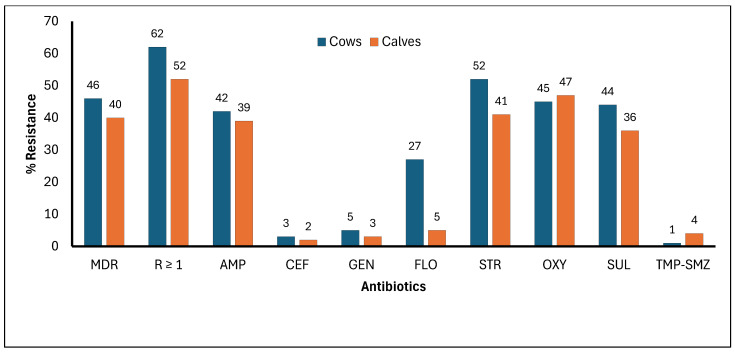
Frequency of fecal *Escherichia coli* resistance to eight antibiotics in beef cow–calf operations (comparison between cows and calves) (MDR = Multi-drug resistance; R ≥ 1 = Resistance to one or more antibiotics; AMP = Ampicillin; CEF = Ceftiofur; GEN = Gentamicin; FLO = Florfenicol; STR = Streptomycin; OXY = Oxytetracycline; SUL = Sulfadimethoxine; TMP-SMZ = Trimethoprim–sulfamethoxazole).

The frequency of cows and calves resistant to one or more antibiotics was 62% (23 + 57 + 170 = 250/403) and 52% (15 + 37 + 100 = 152/292), respectively (Figure 2 and Table S2). Similarly, MDR was identified in 46% (18 + 32 + 137 = 187/403) of cows and 40% (11 + 34 + 71 = 116/292) of calves.

### 3.4. Relationship Between Farm Size (Small, Medium, and Large), Animal Type (Cows and Calves), and AMR

The odds of *E. coli* resistance to one or more antibiotics were higher in medium and large beef cow–calf operations, compared to small beef cow–calf operations with odds ratios (OR) of 1.67 (95% CI = 1.28–2.13) and 2.70 (95% CI = 1.49–5.00), respectively (Table 2). For individual antibiotics, the odds of resistance to streptomycin and sulfadimethoxine were also higher in medium and large beef cow–calf operations, compared to small beef cow–calf operations. For streptomycin, the ORs were 1.92 (95% CI = 0.96–4.00) and 4.17, 95% CI = 2.50–6.67, respectively; for sulfadimethoxine, ORs were 1.64 (95% CI = 1.03–2.63) and 3.45 (95% CI = 2.56–4.76), respectively.

Additionally, the odds of *E. coli* resistance to florfenicol were higher in calves than in cows, after controlling for farm size (OR = 3.57, 95% CI = 1.22–11.11) (Table 2).

## 4. Discussion

This study, which employed phenotypic AST, provides new insights into the frequency and comparative patterns of antimicrobial-resistant fecal *E. coli* in cows and calves housed in small, medium, or large beef cow–calf operations in Florida.

The odds of *E. coli* resistance to one or more antibiotics were higher in medium and large beef cow–calf operations, compared to small beef cow–calf operations. Possible explanations for the higher resistance observed in large or medium cow–calf operations include greater antimicrobial selective pressure, resulting from more frequent antibiotic use for therapeutic purposes. For example, ranchers from large beef cow–calf operations informally reported using tetracyclines, florfenicol, and penicillin-class drugs to treat diarrhea, respiratory diseases, foot rot, and parasitic infestations (anaplasmosis). Consistent with this, the 2017 NAHMS beef study found that 82.9% of large and 79.8% of medium-sized operations used antibiotics to treat at least one animal, compared with only 42.1% of small operations [11]. Furthermore, larger grazing areas and higher animal populations may contribute to increased environmental contamination and contact with wildlife, enhancing the environmental reservoir and transmission of resistance genes. Cattle in large and medium operations often share pastures with rodents, feral swine, and other wildlife, allowing direct or indirect (soil or water) transmission of resistant microbes. Studies from beef cow–calf operations in Florida have shown that the gut microbiota and resistance gene profiles of cattle overlap with those of feral swine and coyotes in shared grazing zones, suggesting cross-species transmission of antimicrobial-resistant bacteria and genes [22]. Additionally, large and medium-sized operations are typically more commercially oriented, involving increased animal transportation, mixing animals prior to shipment, and greater stress during calving and weaning seasons—all factors that can facilitate the dissemination of resistant bacteria [23,24,25]. Our findings align with those of Markland et al. [12] and Ferroni et al. [15], who also reported higher AMR in large beef cow–calf operations. Markland et al. [12], studying 17 beef cow–calf farms in Florida, United States, found that herds with more than 500 cattle exhibited greater cefotaxime resistance, whereas Ferroni et al. [15], investigating 54 cow–calf farms in Italy, observed that larger farms (>100 animals) had a significantly higher prevalence of ESBL-/AmpC-producing *E. coli* than smaller farms. Although direct comparison is limited, due to differences in herd numbers, animal type, and farm size classification, as well as the antibiotic testing panel, these studies collectively support the hypothesis that larger beef cow–calf operations tend to exhibit higher antimicrobial resistance.

The odds of resistance to streptomycin and sulfadimethoxine were higher in medium and large beef cow–calf operations, compared to small operations. A trend was observed for streptomycin resistance, with higher resistance in medium and large operations, possibly reflecting the historical use of aminoglycosides in beef cow–calf systems or cross-resistance resulting from the use of other aminoglycoside compounds [26]. Although streptomycin is no longer widely used in modern veterinary practice, resistance may persist through co-selection mechanisms, as aminoglycoside resistance genes are often located on mobile genetic elements that also harbor genes conferring resistance to other antimicrobial classes [26]. Some aminoglycosides remain approved for livestock use under prescription-only restrictions, which may lead to the development of selective pressure in certain herds. In addition to streptomycin, resistance to sulfadimethoxine was also higher in medium and large operations. This may also reflect the past use of sulfonamides for therapeutic and metaphylactic purposes in cattle. Although sulfonamide use has declined in recent years, resistance genes can persist within bacterial populations through co-selection and horizontal gene transfer. Antibiotic residues and resistant bacteria excreted in urine and feces can persist in soil and manure for extended periods, serving as environmental reservoirs for re-exposure and resistance dissemination [27]. The soil microbiota itself may represent a potential source of resistant organisms in cattle [28], especially in pasture-based operations, such as those examined in this study. Moreover, well-documented cross-resistance among sulfonamide compounds further complicates efforts to attribute resistance to a single drug [29]. In Canada, Carson et al. [30] and Waldner et al. [31] observed low levels of *E. coli* resistance to streptomycin in beef cow–calf operations, while Gow et al. [32,33] reported substantially higher rates of 42% (86/207) and 67% (71/106), respectively. Another study by Gow et al. [24] found 49% streptomycin resistance in calves sampled in the spring but only 5% in those sampled in the fall. For sulfadimethoxine, Morris et al. [17] reported 25% (62/244) *E. coli* resistance in beef cow–calf operations at the isolate level, while Abdelfattah et al. [34] documented 32% resistance in dairy cattle in California, United States. Direct comparison with our findings is limited, as these studies employed different AMR detection methods (e.g., broth microdilution), reported resistance at the isolate level rather than the animal level, and involved different production systems (e.g., dairy versus beef cattle). However, to our knowledge, no previous study has compared resistance to these antimicrobials among beef cow–calf operations of different sizes. Taken together, these results suggest that even with limited or discontinued use of streptomycin and sulfonamides, resistance to these antimicrobial agents persists in beef cow–calf operations.

The odds of *E. coli* resistance to florfenicol were higher in calves, compared with cows, after controlling farm size. A likely explanation is the frequent use of florfenicol to manage infectious keratoconjunctivitis, bacterial pneumonia, respiratory infections, and pododermatitis [35,36,37]. This therapeutic practice may contribute to the higher florfenicol resistance observed among calves, as several ranchers in this study informally reported using florfenicol for treating respiratory infections in calves. In addition, resistance to florfenicol is typically mediated by mobile genetic elements, such as plasmids and integrons, which facilitate horizontal transfer of resistance genes among bacterial populations [38], promoting rapid dissemination of resistance within and between microbial communities.

Although no previous study has directly compared florfenicol resistance between calves and cows, earlier research has shown that calves often harbor higher levels of antimicrobial-resistant *E. coli* than adult cattle. For example, Berge et al. [14] and Gow et al. [39] reported higher overall antimicrobial resistance and higher tetracycline resistance in calves, compared to cows, respectively. Similarly, a study of dairy farms in Puerto Rico detected the *floR* gene in 63% of *E. coli* isolates from calves but only in 13% from cows, suggesting that young animals may serve as important reservoirs of florfenicol-resistant strains [40]. However, that study was based on a relatively small number of isolates. Direct comparison with these studies is limited due to differences in herd and animal numbers, as well as the methods used for AMR detection. Nonetheless, the collective evidence supports the hypothesis that *E. coli* isolated from calves exhibit higher levels of resistance than those from adult cows.

This study has several limitations. First, although the number of animals sampled within each farm was representative of the study population in that farm, a random selection of the study population was not feasible, due to inherent logistical challenges, particularly on large farms. Second, formal records of antimicrobial use in the study herds were unavailable, limiting our ability to directly assess the relationship between antimicrobial use frequency and AMR. Finally, although the nine study farms represent the most common livestock management practices in small, medium, and large cow–calf operations in Florida, the findings cannot be generalized to all cow–calf operations in Florida or beyond.

Despite these limitations, our study’s results provide valuable information to support education and outreach efforts targeting stakeholders, including producers, attending veterinarians, and extension specialists, on best practices for antimicrobial use, particularly within medium- and large-scale cow–calf operations. In addition, our results showed that the burden of AMR, particularly to streptomycin and sulfadimethoxine, was higher in medium and large beef cow–calf operations. However, this finding needs to be confirmed in future studies, involving a larger number of beef cow–calf operations, a broader antibiotic panel, and more advanced molecular techniques, such as next generation sequencing (metagenome, resistome, and mobilome analyses). This will allow us to better characterize resistance mechanisms and transmission pathways.

## 5. Conclusions

Resistance to at least one antibiotic, as well as to streptomycin and sulfadimethoxine, was higher in large and medium operations, compared with small operations. Additionally, calves demonstrated higher resistance to florfenicol than cows. These findings indicate that both operation size and animal age group significantly influence the burden of antimicrobial resistance in beef cow–calf operations.

## Figures and Tables

**Table 1:** Number of cows and calves sampled and tested from each beef cow–calf operation.

Beef Cow–Calf Operations	Size	Number of Samples Collected		Number of Samples Tested for AST	
		Cows	Calves	Cows	Calves
#1	Small	22	11	20	10
#2		18	9	18	9
#3		19	18	18	17
#4	Medium	48	29	42	26
#5		44	30	40	28
#6		32	30	31	28
#7	Large	79	62	76	58
#8		82	65	78	61
#9		85	60	80	55
**Total**		**429**	**314**	**403**	**292**
		**743**		**695**	

**Table 2:** Relationship between farm size (small, medium, large) and animal type (cows, calves) in association with AMR.

Antibiotic	Variable	Category	Odds Ratio (OR)	95% CI	*p*-Value
Multi-drug resistance	Farm size	Small	1.00	-	NA
		Medium	1.19	0.55–2.56	0.602
		Large	2.17	1.14–4.00	0.023
	Animal type	Cow	1.00	-	NA
		Calf	1.05	0.64–1.75	0.797
R ≥ 1 antibiotic	Farm size	Small	1.00	-	NA
		Medium	1.67	1.28–2.13	0.002
		Large	2.70	1.49–5.00	0.006
	Animal type	Cow	1.00	-	NA
		Calf	1.15	0.81–1.61	0.3569
Ampicillin	Farm size	Small	1.00	-	NA
		Medium	1.06	0.69–1.61	0.753
		Large	1.20	0.62–2.38	0.509
	Animal type	Cow	1.00	-	NA
		Calf	0.99	0.81–1.23	0.954
Ceftiofur *	Farm size	Small	1.00	-	NA
		Medium	ND	ND	ND
		Large	ND	ND	ND
	Animal type	Cow	1.00	-	NA
		Calf	ND	ND	ND
Gentamicin	Farm size	Small	1.00	-	NA
		Medium	0.44	0.20–0.99	0.048
		Large	0.23	0.08–0.60	0.010
	Animal type	Cow	1.00	-	NA
		Calf	1.64	0.51–5.56	0.342
Florfenicol	Farm size	Small	1.00	-	NA
		Medium	0.80	0.05–11.11	0.844
		Large	4.00	1.19–12.50	0.031
	Animal type	Cow	1.00	-	NA
		Calf	3.57	1.22–11.11	0.028
Streptomycin	Farm size	Small	1.00	-	NA
		Medium	1.92	0.96–4.00	0.062
		Large	4.17	2.50–6.67	<0.001
	Animal type	Cow	1.00	-	NA
		Calf	1.41	0.86–2.27	0.137
Oxytetracycline	Farm size	Small	1.00	-	NA
		Medium	1.54	0.60–4.00	0.309
		Large	2.44	1.08–5.26	0.035
	Animal type	Cow	1.00	-	NA
		Calf	0.67	0.27–1.64	0.314
Sulfadimethoxine	Farm size	Small	1.00	-	NA
		Medium	1.64	1.03–2.63	0.042
		Large	3.45	2.56–4.76	<0.001
	Animal type	Cow	1.00	-	NA
		Calf	1.29	0.86–1.96	0.169
Trimethoprim–sulfamethoxazole	Farm size	Small	1.00	-	NA
		Medium	1.45	0.14–14.3	0.712
		Large	0.47	0.05–4.76	0.453
	Animal type	Cow	1.00	-	NA
		Calf	0.49	0.14–1.79	0.225

* Ceftiofur did not converge in SAS; NA = not applicable; ND = not determined; OR = odds ratio; CI = confidence interval.

## Data Availability

The original contributions presented in this study are included in the article/Supplementary Materials. Further inquiries can be directed to the corresponding author.

## Supplementary Materials

The following supporting information can be downloaded at: https://www.mdpi.com/article/10.3390/microorganisms14010013/s1. Table S1: Frequency of fecal *Escherichia coli* resistance to eight antibiotics among cattle from small, medium, and large beef cow–calf operations. Table S2: Frequency of fecal *Escherichia coli* resistance to eight antibiotics in beef cow-calf operations (comparison between cows and calves).
