# Supplementary material for: Frequency of Antimicrobial-Resistant Fecal Escherichia coli Among Small, Medium, and Large Beef Cow–Calf Operations in Florida
Source: Microorganisms. 2025 Dec 20;14(1):13. doi: 10.3390/microorganisms14010013 (PMC12844052; doi:10.3390/microorganisms14010013)
Supplement: Supplementary file 1 [file microorganisms-14-00013-s001.zip › microorganisms-4028516-supplementary.pdf]

Table S1. Frequency of fecal *Escherichia coli* resistance to eight antibiotics among cattle from small, medium, and large beef cow–calf operations.

	Small Cow-calf operations			Medium Cow-calf operations			Large Cow-calf operations			Total
	#1 n/N (%)	#2 n/N (%)	#3 n/N (%)	#4 n/N (%)	#5 n/N (%)	#6 n/N (%)	#7 n/N (%)	#8 n/N (%)	#9 n/N (%)	
**Multi-drug resistance**	13/30 (43)	7/27 (26)	9/35 (25)	18/68 (26)	21/68 (31)	28/59 (47)	63/134 (47)	78/139 (56)	67/135 (50)	**304/695 (44)**
	**29/92 (32)**			**67/195 (34)**			**208/408 (51)**			
**Resistance to ≥ 1 antibiotics**	13/30 (43)	11/27 (41)	14/35 (40)	33/68 (49)	36/68 (53)	35/59 (59)	91/134 (68)	105/139 (76)	74/135 (55)	**412/695 (59)**
	**38/92 (41)**			**104/195 (53)**			**270/408 (66)**			
**Ampicillin**	12/30 (40)	8/27 (30)	14/35 (40)	31/68 (46)	22/68 (32)	21/59 (36)	57/134 (43)	77/139 (55)	41/135 (30)	**283/695 (41)**
	**34/92 (37)**			**74/195 (38)**			**175/408 (43)**			
**Ceftiofur**	0/30 (0)	0/27 (0)	0/35 (0)	0/68 (0)	4/68 (6)	0/59 (0)	1/134 (1)	13/139 (9)	1/135 (1)	**19/695 (3)**
	**0/92 (0)**			**4/195 (2)**			**15/408 (4)**			
**Gentamicin**	4/30 (13)	2/27 (7)	2/35 (6)	2/68 (3)	4/68 (6)	2/59 (3)	7/134 (5)	2/139 (1)	2/135 (1)	**27/695 (4)**
	**8/92 (9)**			**8/195 (4)**			**11/408 (3)**			
**Florfenicol**	2/30 (7)	1/27 (4)	2/35 (6)	1/68 (1)	0/68 (0)	11/59 (19)	15/134 (11)	38/139 (27)	56/135 (41)	**126/695 (18)**
	**5/92 (5)**			**12/195 (6)**			**109/408 (27)**			
**Streptomycin**	9/30 (30)	7/27 (26)	7/35 (20)	18/68 (26)	24/68 (35)	30/59 (51)	79/134 (59)	86/139 (62)	70/135 (52)	**330/695 (47)**
	**23/92 (25)**			**72/195 (37)**			**235/408 (58)**			
**Oxytetracycline**	11/30 (37)	9/27 (33)	7/35 (20)	18/68 (26)	24/68 (35)	35/59 (59)	54/134 (40)	92/139 (66)	69/135 (51)	**319/695 (46)**
	**27/92 (29)**			**77/195 (39)**			**215/408 (53)**			
**Sulfadimethoxine**	8/30 (27)	6/27 (22)	7/35 (20)	21/68 (31)	16/68 (24)	24/59 (41)	64/134 (48)	79/139 (57)	66/135 (49)	**291/695 (42)**
	**21/92 (23)**			**61/195 (31)**			**209/408 (51)**			
**Trimethoprim/ Sulfamethoxazole**	2/30 (7)	0/27 (0)	0/35 (0)	1/68 (1)	2/68 (3)	8/59 (14)	2/134 (1)	1/139 (1)	2/135 (1)	**18/695 (3)**
	**2/92 (2)**			**11/195 (6)**			**5/408 (1)**			

Table S2: Frequency of fecal *Escherichia coli* resistance to eight antibiotics in beef cow-calf operations (comparison between cows and calves)

	Small Cow-calf operations						Medium Cow-calf operations						Large Cow-calf operations					
	Cows			Calves			Cows			Calves			Cows			Calves		
	#1	#2	#3	#1	#2	#3	#4	#5	#6	#4	#5	#6	#7	#8	#9	#7	#8	#9
**Multi-drug resistance**	8/20 (40)	5/18 (28)	5/18 (28)	5/10 (50)	2/9 (22)	4/17 (24)	10/42 (24)	8/40 (20)	14/31 (45)	8/26 (31)	13/28 (46)	14/28 (50)	32/76 (42)	51/78 (65)	54/80 (68)	31/58 (53)	27/61 (44)	13/55 (24)
	**18/56 (32)**			**11/36 (31)**			**32/113 (28)**			**34/82 (41)**			**137/234 (59)**			**71/174 (41)**		
**Resistance to ≥ 1 antibiotics**	8/20 (40)	8/18 (44)	7/18 (39)	5/10 (50)	3/9 (33)	7/17 (42)	20/42 (48)	18/40 (45)	19/31 (61)	13/26 (50)	18/28 (64)	16/28 (57)	54/76 (71)	61/78 (78)	55/80 (69)	37/58 (63)	44/61 (72)	19/55 (35)
	**23/56 (41)**			**15/36 (42)**			**57/113 (50)**			**37/82 (45)**			**170/234 (73)**			**100/174 (57)**		
**Ampicillin**	8/20 (40)	5/18 (28)	7/18 (39)	4/10 (40)	3/9 (33)	7/17 (41)	19/42 (45)	10/40 (25)	10/31 (32)	12/26 (46)	12/28 (43)	11/28 (39)	33/76 (43)	49/78 (63)	29/80 (36)	24/58 (41)	28/61 (46)	12/55 (22)
	**20/56 (36)**			**14/36 (39)**			**39/113 (35)**			**35/82 (43)**			**110/234 (47)**			**64/174 (37)**		
**Ceftiofur**	0/20 (0)	0/18 (0)	0/18 (0.0)	0/10 (0.0)	0/9 (0)	0/17 (0)	0/42 (0)	1/40 (2.5)	0/31 (0)	0/26 (0)	3/28 (11)	0/28 (0)	0/76 (0)	12/78 (15)	1/80 (1)	1/58 (2)	1/61 (2)	0/55 (0)
	**0/56 (0)**			**0/36 (0)**			**1/113 (1)**			**3/82 (4)**			**13/234 (6)**			**2/174 (1)**		
**Gentamicin**	2/20 (10)	2/18 (11)	1/18 (6)	2/10 (20)	0/9 (0)	1/17 (6)	1/42 (2)	3/40 (8)	1/31 (3)	1/26 (4)	1/28 (4)	1/28 (4)	7/76 (9)	0/78 (0)	2/80 (3)	0/58 (0)	2/61 (3)	0/55 (0)
	**5/56 (9)**			**3/36 (8)**			**5/113 (4)**			**3/82 (4)**			**9/234 (4)**			**2/174 (1)**		
**Florfenicol**	1/20 (5.00)	1/18 (6)	2/18 (17)	1/10 (10)	0/9 (0)	0/17 (0.0)	1/42 (2)	0/40 (0)	6/31 (19)	0/26 (0)	0/28 (0)	5/28 (18)	10/76 (13)	37/78 (47)	52/80 (65)	5/58 (9)	1/61 (2)	4/55 (7)
	**4/56 (7)**			**1/36 (3)**			**7/113 (6)**			**5/82 (6)**			**99/234 (42)**			**10/174 (6)**		
**Streptomycin**	6/20 (30)	5/18 (28)	5/18 (28)	3/10 (30)	2/9 (22)	2/17 (12)	12/42 (29)	9/40 (23)	16/31 (52)	6/26 (23)	15/28 (54)	14/28 (50)	47/76 (62)	55/78 (71)	55/80 (69)	32/58 (55)	31/61 (51)	15/55 (27)
	**16/56 (29)**			**7/36 (19)**			**37/113 (33)**			**35/82 (43)**			**157/234 (67)**			**78/174 (45)**		
**Oxytetracycline**	6/20 (30)	7/18 (39)	0/18 (0)	5/10 (50)	2/9 (22)	7/17 (41)	7/42 (17)	10/40 (25)	19/31 (61)	11/26 (42)	14/28 (50)	16/28 (57)	20/76 (26)	57/78 (73)	55/80 (69)	34/58 (59)	35/61 (57)	14/55 (25)
	**13/56 (23)**			**14/36 (39)**			**36/113 (32)**			**41/82 (50)**			**133/234 (57)**			**82/174 (47)**		
**Sulfadimethoxine**	6/20 (30)	4/18 (22)	4/18 (44)	2/10 (20)	2/9 (22)	3/17 (18)	12/42 (29)	8/40 (20)	12/31 (39)	9/26 (35)	8/28 (29)	12/28 (43)	35/76 (46)	50/78 (64)	54/80 (68)	29/58 (50)	29/61 (48)	12/55 (22)
	**14/56 (25)**			**7/36 (19)**			**32/113 (28)**			**29/82 (35)**			**139/234 (59)**			**70/174 (40)**		
**Trimethoprim-sulfamethoxazole**	1/20 (5)	0/18 (0)	0/18 (0)	1/10 (10)	0/9 (0.0)	0/17 (0)	0/42 (0)	1/40 (3)	0/31 (0)	1/26 (4)	1/28 (4)	8/28 (29)	2/76 (3)	1/78 (1)	1/80 (1)	0/58 (0)	0/61 (0)	1/55 (2)
	**1/56 (2)**			**1/36 (3)**			**1/113 (1)**			**10/82 (12)**			**4/234 (2)**			**1/174 (1)**		
